# Surgery in Vukovar War

**DOI:** 10.3325/cmj.2011.52.218

**Published:** 2011-04

**Authors:** Ivica Matoš

**Affiliations:** Department of Urology, Osijek General Hospital, Osijek, Croatia

I was born in Čamagajevci near Donji Miholjac in 1947. My parents moved to Vukovar immediately after my birth so I grew up there. I graduated from the Zagreb University School of Medicine in 1972. I completed my urology residency in the Zagreb Hospital Center in 1980 and obtained my master's degree in biomedicine in 1989. I worked in Vukovar until 1986, when I got a position in the Osijek General Hospital. My family remained in Vukovar and, when the war broke out, I got a permission to stay there and work in the Vukovar hospital.

## Foreword

The data and numbers mentioned in the text are from my memory. No written material is available since the documentation was seized by the Yugoslav Federal Army (YFA) after the fall of Vukovar. I wish Dr Vesna Bosanac, the director of the Vukovar Medical center, and Dr Juraj Njavro, the head of the Vukovar Medical Corps Headquarters, were now with us because they both knew more and had all the relevant documentation. They were captured by the YFA.

## Surgery in war

In the beginning, six general surgeons, three orthopedic surgeons, one urology surgeon, three anesthesiologists, and a few surgery residents worked in the surgery ward. Some left the hospital early in the year, when the unrest in the surrounding villages began. Anesthesiologists from Osijek were called to help us. At the same time, the Croatian Ministry of Health organized mobile surgical teams that came to Vukovar to help for a week or two. Our ward functioned normally because the fights occurred at the periphery of the town. The first air raids started in July, when we rushed to the basement for the first time. Since August 25, 1991, a general danger alert was on the whole time, and the life and work of the medical center took place in the basement and its anti-atom bomb (AA) shelter. When the number of the wounded was below 50, the AA-shelter functioned perfectly, but its increase forced us to work in less safe parts of the hospital. Shelling of the hospital mostly coincided with successes of our soldiers in the battlefield. I am absolutely sure that the YFA “punished” the hospital for every lost battle.

## Electricity

As early as at the beginning of September, the electricity supply did not function properly and we had to activate our own generators. By the end of September, electricity was completely cut off and we relied solely on the generators. As the communication with Zagreb was possible at the time, we asked and got a portable power supply. Dr Bosanac's husband was an engineer in the Borovo tire factory and he and his coworkers managed to run a generator in the factory to supply the vital institutions in the town. This system functioned quite well except for the last 10 days before the take-over of the hospital. Of course, many interruptions and repairs were needed because the YFA knew what to target. In the end, we relied solely on a single small portable generator because the large one was destroyed. Another small generator was used for x-ray service only.

## Water

The problems with electricity were followed by those with water. In September, the town's water supply system was destroyed. The Center had tanks totaling some 2000 L, refilled by water from the cistern trucks from Borovo. Then the road to Borovo was cut off, the trucks were destroyed, and three firemen were killed. For the last few weeks, we brought water from nearby wells under constant artillery fire. Our daily allowance was half a liter of drinking water. Personal hygiene was poor. We were happiest when it rained – we then collected rain water in pots and washed ourselves. The water was regularly checked and chlorinated.

## Surgical theaters

We prepared for the war in time. The theaters for small surgery were adapted for larger surgeries, and the necessary instruments were transferred there. (Still we believed it would never be necessary.) Frequent bombing forced us soon to move to the basement, and doctor's offices were used for small interventions. Soon the number of the wounded drastically increased and it often happened that several heavy wounded waited for a surgery. New theaters had to be improvised: the plaster and one of the x-ray rooms were used. They functioned to the end.

Working conditions were impossible, as the building shook and swayed with explosions. We never stopped the surgeries. The greatest problem was that the wounded often arrived in large numbers, and we sometimes worked kneeling by a patient on a stretcher.

Sterilization of the instruments functioned and was performed mostly during the night, after daily surgeries. In the beginning, we used a big autoclave; later we switched to dry sterilizers because there was not enough water.

Window glass shattered and dust filled the theater after a close explosion. We replaced the glass with plastic sheets. Theaters were also very cold because the heating system was destroyed in September.

In the darkness of the hospital basement, we also lost the sense of time, and it is now difficult to remember most dates.

## Medicines

All patients received anti-tetanus vaccine and antibiotics while we had them. For the last month of the siege, we were short of antibiotics, particularly of the more potent ones. Weaker ones were used and that certainly influenced the outcome of several cases. Some medications were recovered from the physicians' offices in the town. Brave men risked their lives to go to the town and search for medicines. Our stock was soon empty as the average number of the wounded that came to the hospital rose to 30 per day. Once we received 90 wounded in a single day.

## Hospital beds

Patients who would ordinarily be released from the hospital were sometimes kept in if they had no place to go, or if the artillery fire was so intense that they could not leave the building. During the worst of days, the patients lay in pairs in and under the beds. This prompted us to separate lightly wounded and send them to the Borovokomerc shelter where we organized a dislocated medical unit. However, the connection with the unit was soon cut off and all patients had to stay in the hospital.

## Patients

Until November 15, 1991, 800 Croatian National Guardsmen, 838 civilians, 111 policemen, and 7 YFA soldiers were admitted to the hospital. Civilians were of all nationalities, and were treated equally, according to the ethical codes of our profession. It should be emphasized that, in the beginning, most of the wounded were civilians. The age of the wounded ranged from 6 months to 88 years (famous Vukovar's pharmacist, V.S.). Almost 70% of the wounded were in the 20-40 age group. Their moral and the spirit were high. They would start singing during the worst of attacks. Even the most heavily wounded asked when they would return to the battlefront.

## Wounds

Due to very destructive projectiles, wounds were numerous and very serious. Combined wounds of the chest and the abdomen were common. More than 80% of wounds were of explosive origin, and only 10% were gunshot wounds. Approximately 5% were burns, more frequent during the last days, when the YFA used napalm bombs. Other injuries were caused by falling buildings and blast waves. The destructions of soft tissue and bones were often combined. Multiple injuries were mostly caused by fragmentation shells, such as cluster bombs and bullets, forbidden by international agreements.

The quality of the wound treatment was satisfactory. In some dozen cases we prevented the amputation of extremities by vascular surgery, termino-terminal anastomoses, and replacement of a damaged artery by a vein. Bone fractures were replaced by external fixators.

Almost to the very end, we did not observe any significant infections. As the water and sanitation problems increased, gaseous gangrene appeared in 9 patients during the three last weeks. Partial or total amputation of the extremities had to be performed and 3 patients died.

## Other surgical cases

I recorded 11 urogenital operations. In two cases, the bladder was completely destroyed, with accompanying injury of the pelvis and systemic shock. An ureterocutaneous anastomosis had to be performed. Both patients survived and are now treated in Zagreb. I believe that there were many more injuries to the urogenital tract, but the patients did not reach the hospital alive because of other concomitant injuries.

## A case

On admission, the examination of a patient revealed a small entrance gunshot wound on the upper part of the leg. x-ray machine was out of order that day, and we treated the injury according to the principles of the war surgery. After a few hours the patient developed the signs of acute abdomen and was operated. The projectile was found in the upper abdomen, where it damaged internal organs.

## Fall of Vukovar

On November 19, 1991, we were aware that Vukovar had fallen. YFA entered the town, and we were told not to leave the hospital during the night. YFA guarded the hospital, both regular soldiers and Serbian paramilitary forces. Among them there was a number of our neighbors and friends of Serbian nationality. Several members of the hospital staff were taken away that night. The night was otherwise silent, with only faraway detonations.

## Evacuation

The evacuation was organized and supervised by the Serbian Red Cross only. International Red Cross team had spent an hour or two in the hospital yard in the morning. European Commission Mission members just observed the events. In the morning of November20, the medical personnel was ordered to gather in a room at 7:30. A YFA officer announced that from now on military rules were in order, and that Dr Bosanac is relieved of her function as the hospital director. He gave a speech on Yugoslavia and YFA. During that time, as we later learned, ambulant patients were taken away in busses to the YFA base. Several members of the hospital staff and husbands of our female personnel, who came to the hospital to be with their children and wives and helped in the hospital, were also taken away. Upon our protests, a few of them were released.

The remaining patients were loaded into the trucks by the medical personnel. The convoy was formed and headed for Petrijevci. It turned later toward Negoslavci (opposite of the way that was agreed upon by the YFA). We were told that Croatian officials did not want to receive us, and since we had wounded with us we would be heading to Sremska Mitrovica to stay overnight until the negotiations were over. The wounded were transferred to the medical unit of the local garrison. Two of them were soon transferred to Belgrade by a helicopter because of their condition. We slept in the busses because we did not want to be separated.

Next morning, around 11, we headed for Rata near Bijeljina in Bosnia. Banjski Dvori was the place of the transfer to Croatian vehicles. The YFA left us and the Bosnian police took over the control of the convoy. I was in the front of the convoy and did not see what happened behind, where two ambulances were destroyed and wounded and personnel attacked by a group of local citizens and Serbian paramilitary. European Commission delegates intervened and stopped the attack. We felt great joy and relief when we crossed the bridge over the Sava river and finally entered free Croatian territory.

